# Linalool Nanoemulsion Preparation, Characterization and Antimicrobial Activity against *Aeromonas hydrophila*

**DOI:** 10.3390/ijms222011003

**Published:** 2021-10-12

**Authors:** Weiming Zhong, Puyu Tang, Ting Liu, Tianyu Zhao, Jiajing Guo, Zhipeng Gao

**Affiliations:** 1 Hunan Engineering Technology Research Center of Featured Aquatic Resources Utilization, College of Animal Science and Technology, Hunan Agricultural University, Changsha 410128, China; zhongweiming2021@163.com (W.Z.); tangpuyu21@mails.ucas.ac.cn (P.T.); z1583025825@163.com (T.Z.); 2 Hunan Agriculture Product Processing Institute, Hunan Academy of Agricultural Sciences, Changsha 410125, China; ltchangsha98@163.com; 3 Key Laboratory of Agro-Products Processing, Ministry of Agriculture and Rural Affairs of P. R. China, Institute of Food Science and Technology CAAS, Beijing 100193, China

**Keywords:** Linalool, nanoemulsion, *Aeromonas hydrophila*, antibacterial activity

## Abstract

*Aeromonas hydrophila* is one of the most important aquatic pathogens causing huge economic losses to aquaculture. Linalool, a vital ingredient of a variety of essential oils, was proved as a good antimicrobial agent in our previous studies. However, the low solubility and volatility of Linalool obstruct its application in the field of aquatic drugs. Thus, in this study, Linalool nano-emulsion (LN) was prepared to solve these obstructions. We investigated the physicochemical properties, antibacterial activity, and mode of action of LN against *A. hydrophila.* LN with different medium chain triglycerides (MCT) concentrations were prepared by ultrasonic method. The results showed that the emulsion droplet size of LN was the smallest when MCT was not added to the formulation. Nano-emulsions are usually less than 500 nm in diameter. In our study, LN in this formulation were spherical droplet with a diameter of 126.57 ± 0.85 nm and showed good stability. LN showed strong antibacterial activity, the MIC and MBC values were 0.3125% *v*/*v* and 0.625% *v*/*v*, respectively. The bacterial population decreased substantially at 1 × MIC of LN. LN exhibited disruptive effect on cell membranes by scanning electron microscope (SEM) and transmission electron microscope (TEM). The present study provided a formulation of Linalool nano-emulsion preparation. Moreover, the good antibacterial activity of LN showed in our study will promote the application of Linalool for the control and prevention of *A. hydrophila* in aquaculture.

## 1. Introduction

*A. hydrophila* is a gram-negative and zoonotic pathogen widely distributed in various environments, which causes outbreaks of bacterial septicemia resulting in huge losses to aquaculture industry [1,2]. Moreover, *A. hydrophila* may transmitted between humans and animals causing a range of human diseases such as bacteremia [3], gastroenteritis [4], necrotizing fasciitis [5], and so on. Currently, diseases caused by *A. hydrophila* are mainly treated with antibiotics. However, the excessive use and abuse of antibiotics usually causes drug residues, environmental pollutions, and the emergence of drug-resistant bacteria [6,7]. Therefore, it is extremely urgent to look for new antibacterial agents, especially natural or plant-derived agents, to deal with these problems. Thus, finding new natural antibacterial agents has been the focus of interest in recent years.

Essential oils are derived from natural plant tissues and have the advantages of being eco-friendly, widely available, and easily extractable. Many studies, including our previous studies, have shown that essential oils exhibited strong antibacterial activities [8,9,10,11,12]. Linalool (3,7-dimethyl-1,6-octadien-3-ol) is a vital constituent found in many essential oils with good antibacterial activity against different kinds of microorganisms [13], such as *Listeria monocytogenes* (our previous study) [14], *Pseudomonas fluorescens* [15], *P.aeruginosa* [16], *Salmonella senftenberg* [17], and *S.putrefaciens* [18] etc. It is generally recognized as safe (GRAS) as a food additive. However, the application of Linalool or essential oils is challenged and limited due to high volatility, low utilization rate, and solubility [19].

Nanotechnology is a multidisciplinary technology including nano-chemistry, nano-physics, nano-materials, and nano-biology, etc., which are widely used in the fields of materials [20], electronics [21], and medical [22]. Nano-emulsion is a dispersion system of nanoscale formed by spontaneous emulsification or high-energy emulsification of oil, water, surfactants, etc., which are often used in biology and pharmacology due to its higher solubility and physical stability, lower volatility and turbidity compared with the original substance itself [23]. Meanwhile, several nano-emulsions of essential oils showed significantly better antibacterial activity than their pure essential oils [24,25]. Thus, in the present study, the Linalool nano-emulsion was prepared to solve those obstructions mentioned above. Furthermore, the physicochemical properties and the antibacterial activity of LN against *A. hydrophila* were evaluated.

## 2. Results

### 2.1. The Diameter of LN with Different MCT Concentrations

**Figure 1:**
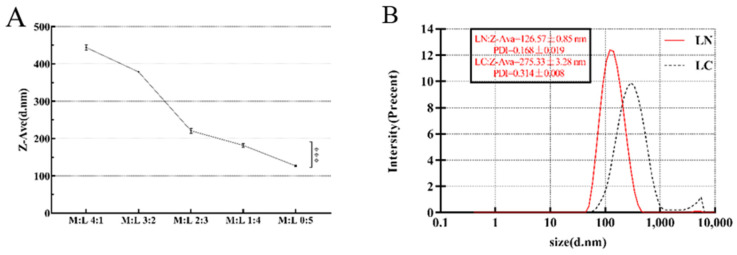
Effect of MCT concentration on the emulsion droplet size of LN. (**A**) Z-Ava values (d. nm) of LN at various M:L (4:1, 3:2, 2:3,1:4 and 0:5) ratios. *** represented *p* < 0.001. M:L represented for MCT: Linalool. (**B**) Z-Ava value (d. nm) and PDI value of LN and LC at the ratio of M:L (0:5).

Figure 1A showed the influence of the emulsion droplet size of LN with different MCT concentrations. The emulsion droplet size decreased with the decreasing concentration of MCT in trends of 443.77 ± 3.89, 378.13 ± 0.75, 220.63 ± 3.76, 181.60 ± 2.81, and 126.57 ± 0.85 nm. In particular, the diameters of the emulsion droplets with and without MCT were significantly different, indicating that MCT significantly increased the emulsion droplet size. Therefore, the best LN formulation was chosen without the addition of MCT. As shown in Figure 1B, LN and linalool colostrum (LC) had different characteristics. The Z-Ava of LN is 126.57 ± 0.85 nm, which was significantly lower than that of LC, the lower polydispersity index (PDI) value of LN indicated a better homogeneity.

### 2.2. Structural, Thermal Stability and Morphological Observations of LN

**Figure 2:**
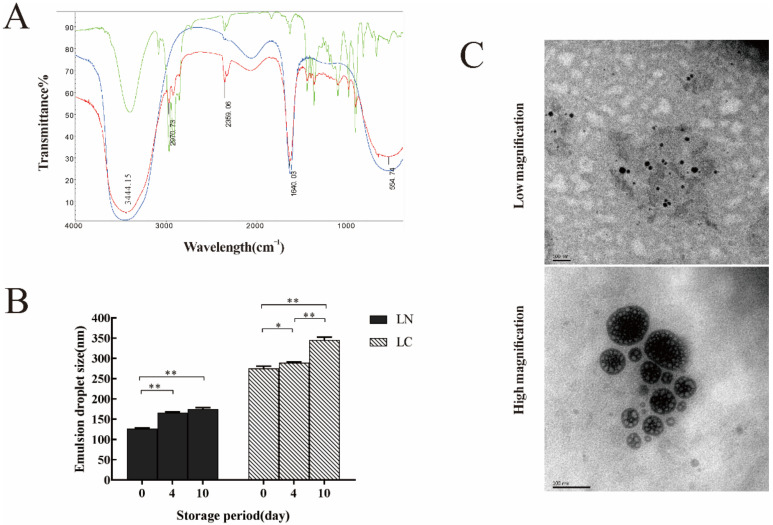
Physicochemical properties of LN. (**A**) Infrared spectra of the Linalool, aqueous phase, and LN by FTIR. Red curve: LN; blue curve: aqueous phase; green curve: Linalool. (**B**) The diameters of LN and LC (nm) were measured after 0, 4, and 10 d of storage. * and ** represented *p* < 0.05 and *p* < 0.01, respectively. (**C**) The TEM observation of the morphology and emulsion droplet size LN.

Fourier Transform Infrared (FTIR) spectroscopy was used to find out functional groups in Linalool, aqueous phase, and LN to determine whether functional groups changed during the formation of LN (Figure 2A). Firstly, the presence of -OH (3441.15 cm^−1^) proved that water was a part of the component of LN. Secondly, the absorption peaks on LN were highly coincident with Linalool. For instance, the absorption peak at 2970.3 cm^−1^ was formed by the stretching vibration of -CH_2_, which was the same as the absorption peak on Linalool. Thirdly, 2359.06 cm^−1^ was the absorption peak due to the effect of CO_2_ during the measurements. Linalool proved that no chemical reaction took place during the preparation of the nano-emulsion.

The thermal stability is an important parameter of nano-emulsion. As shown in Figure 2B, the diameter of LN and LC were measured after 0, 4, and 10 d of storage. No significant phase delamination was observed in any of the samples during storage. The diameter of LN after 4 and 10 d of storage were significantly different from that of 0 d (*p* < 0.01), while there was no significant difference between 4 and 10 d, which indicated that the emulsion droplet size tended to stabilize with time. LC appeared to be in a different situation, the emulsion droplet diameter were all significantly different after 0, 4, and 10 d of storage, and the droplets’ diameter showed a gradual increase with time. Thus, the long-term stability of LN was better than that of LC.

Furthermore, the emulsion droplet size and morphology of LN were observed by TEM as shown in Figure 2C. Most of the droplets of LN were spherical when observed in low magnification, LN showed the emulsion droplet size range of around 120 nm, which was an acceptable nanometer size diameter and was consistent with the size data obtained by Zetasizer Nano ZS90. Further observation through high magnification revealed that LN were not a mere spherical structure, but a spherical substance formed by several small spherical packages.

### 2.3. Analysis of the Antibacterial Activity of LN against A. hydrophila

**Figure 3:**
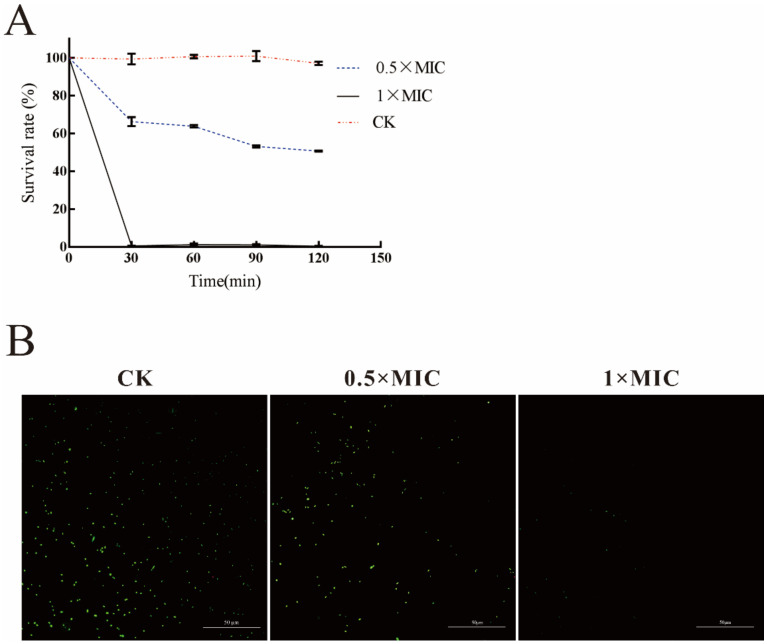
Antimicrobial activity of LN against *A. hydrophila.* (**A**) Bactericidal activity of LN at different treated concentrations. (**B**) Microscopic observation of *A. hydrophila* stained by SYTO^®^9 at different treated concentrations of LN.

The results of antimicrobial concentration assay showed that the MIC and MBC of LN were 0.3125% *v*/*v* and 0.625% *v*/*v*, respectively. As shown in Figure 3A, most of bacteria were killed after 30 min treatment with LN at 1 × MIC concentration. In contrast, the bacterial survival rate was about 50% after treated with 0.5 × MIC LN. SYTO^®^9 is a live cell fluorescent stain, and live cells appear green fluorescent when stained. In Figure 3B, the green fluorescence in the CK, 0.5×MIC, and 1×MIC images decreased with the increasing concentrations of LN, which indicated the significant decrease of the bacterial number.

### 2.4. SEM Observation

**Figure 4:**
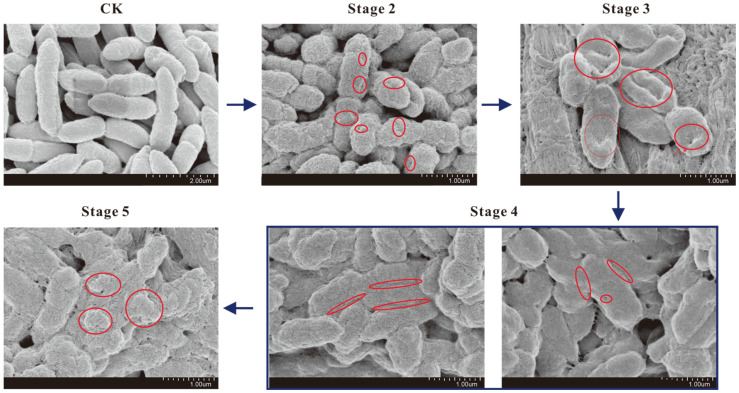
Changes of morphology of *A. hydrophila* at different treatment stages of LN observed by SEM. The red circles indicated the damage of cell membrane.

As shown in Figure 4, the morphological changes of *A. hydrophila* at different treatment stages were evaluated by SEM. In the control group (CK), cells were round-terminated with a length of 1–2 µm, the bacterial surface was rough and intact with clear demarcation between individuals. In stage 2, when the treatment time was 10 min, the bacteria showed various degrees of damage, indicating that the outer membrane of the bacteria was beginning to be disrupted. In stage 3, due to the continuous action of LN, the area of collapse increased, and hollows appeared and cells became flattened, which might be caused by the large amount of cellular materials penetrating outside. In stage 4, the loss of intracellular materials was irreversible and fatal, the outer membrane of individual bacteria began to bond to each other, and the boundaries between cells began to blur. In stage 5, the outer membrane of cells ruptured, and the morphology of cells contracted.

### 2.5. TEM Observation

**Figure 5:**
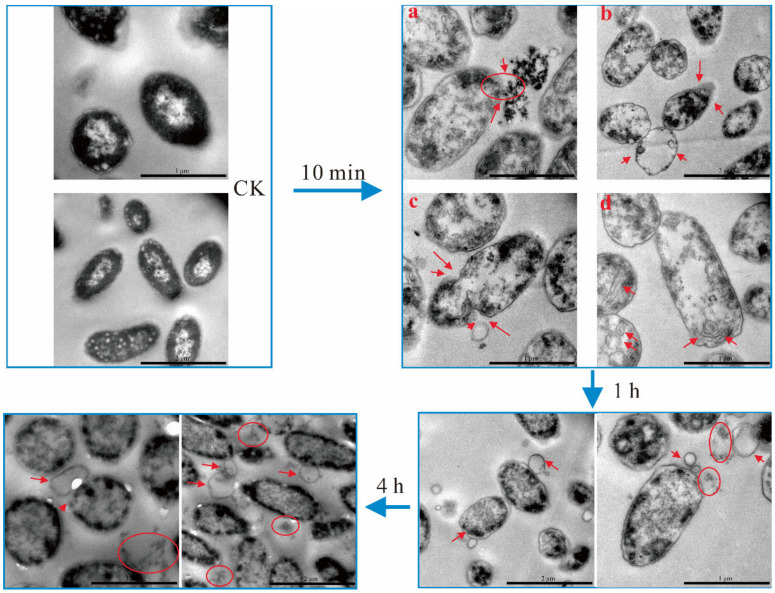
Changes of morphology and cellular materials of *A. hydrophila* after 10 min, 1 h, and 4 h treatment of LN observed by TEM. The red arrows indicated the damage of cell membrane, the red circles indicated intracellular material of cells.

The morphology and cellular materials of *A. hydrophila* at different treatment times were observed by TEM as shown in Figure 5. In the control group (CK), *A. hydrophila* was intact and the cell contents were evenly distributed. After 10 min treatment, cells were visibly swelling, and the cell contents were irregularly distributed. As shown in Figure 5, the intracellular materials were leaking through the cell membrane (10 min-a), and a partial loss of cell contents was observed (10 min-b). Evenly, some of the cell contents were found to be completely lost (10 min-b). Due to the loss of intracellular material, the outer membrane started to sink inwards (10 min-c). Interestingly, many different morphological strips were observed (10 min-d), which might be the fold of cell membrane. When the treatment time increased to 1 h, the cell surface became smooth and intracellular material continued to spill out, while bubbles of varying sizes were derived from cell wall. When the treatment time reached 4 h, the bubble-like structures on the cell wall continued to increase in size and the outer membrane of the cells became blurred.

## 3. Discussion

Aquaculture is the process of artificially breeding aquatic organisms, in which bacterial diseases may significantly reduce the profitability [26,27]. Antibiotics are usually used to deal with bacterial diseases in aquaculture, but due to the causing of drug-resistant bacteria, potential environmental problems, etc., many countries have formulated a series of policies to ban the use of several antibiotics in aquaculture [7]. Thus, the search for alternatives to antibiotics has become an urgent project, and essential oils and their components were noticed by many scholars due to their good antibacterial activity. Many studies were currently focused on food [28] or human [29] pathogens, but litter research had been done for pathogens of aquatic species. The bottlenecks for their use in aquaculture mainly include their low solubility in water and high volatility [30]. In this study, a kind of nanotechnology, nano-emulsion technology, was performed to tackle these obstacles. Many previous studies have proved that MCT was a vital component as emulsion stabilizer to improve the stability of nano-emulsions. *Pimpinella anisum* oil nano-emulsion was prepared under MCT variant, and the diameter of the nano-emulsion was significantly reduced when MCT reached the optimum ratio [31]. At the same time, MCT was the best formulation component in the preparation of the curcumin nano-emulsion [32]. Therefore, our study investigated the effect of different concentrations of MCT on LN formation in order to find the best formulation. However, our results were different from some of the previous studies, MCT had a negative effect on the LN emulsion droplet size. We speculated that a new LN composition may be formed after the addition of MCT, and this composition did not show excellent stability. Our results also indicated that MCT was not always an effective component in the preparation of nano-emulsions.

Although MCT was absent in our formulations, LN still demonstrated good thermodynamic stability. The solubility of LN altered, but not its chemical structure. Many researchers have explored different ways to make different kinds of nano-emulsions. It was demonstrated that MCT, Tween 80, and Linalool in a certain ratio could be used to prepare nano-emulsion by spontaneous emulsification, and the average diameter of the resulting emulsions was 103.24 ± 3.31 nm [33]. However, nanoscale emulsion droplet diameters and good stability were also observed in the mixed nano-emulsion containing Linalool prepared by the microfluidic pressure method [34]. In our study, the colostrum was split into smaller droplets by ultrasonic treatment. When the emulsion droplet diameter is smaller, the Brownian motion of the emulsion droplets in suspension is usually sufficient to counteract the kinetic instability caused by gravity or viscosity. According to previous studies, nano-emulsions are generally spherical droplet [23]. However, it was found by TEM in our study that the spherical shape of the nano-emulsion was formed by numerous blank spherical wraps. We speculated that this spherical LN structure may be a spherical honeycomb formed by the encapsulation of modified soy phospholipids.

Our study showed that LN have strong antibacterial activity against *A. hydrophila*. It was reported that Linalool nano-emulsion exhibited antibacterial activity against *Escherichia coli* O157:H7 and *S. enterica*, LN has the characteristics of high sterilization efficiency and fast sterilization speed. The reason could be that nanotechnology can increase drug delivery, allowing drugs to pass more easily through the extracellular membrane into the cell [35]. *Thymus daenensis* essential oil nano-emulsion showed significantly better antibacterial activity against *E. coli* than pure essential oil, causing massive leakage of intracellular potassium, proteins, and nucleic acids in a short period of time [25]. Similarly, the antimicrobial activities of nano-emulsions (lemongrass, thyme, peppermint, cinnamon, and clove) were higher than that of the essential oils themselves because of the small emulsion droplet size and increased contact area of nano-emulsion [24]. However, savory and thyme essential oils nano-emulsions exhibited no better antibacterial activity than pure essential oils against foodborne pathogens [36]. Therefore, the preparation of nano-emulsion is not always able to improve the antibacterial effect of the drug. It was found in our study that LN was highly destructive to the outer membrane of *A. hydrophila* and the leakage of large amounts of intracellular contents were observed by TEM. This disruption of the cell membrane is a damaging process for the bacteria, meaning that the normal material transport function of the bacterial membrane is unable to function properly [37]. From the observation of SEM and TEM, we hypothesized that when the cell wall and membrane were damaged by LN, the intracellular material was first discharged outwards causing the cells to collapse, and after a period of time, the external water started to enter the cells causing the cells to swell, and finally the cells were completely deformed due to the loss of water. Due to this antibacterial mechanism, LN should be used as a promising antibacterial agent.

## 4. Materials and Methods

### 4.1. Materials

Linalool, surfactant modified soybean phospholipid (MSP), and medium chain triglycerides (MCT) were purchased from Sigma-Aldrich (St. Louis, MA, USA), Henan Siwei Biotechnology Co., Ltd. (Henan, China), and Usolf Chemical Technology Co., Ltd. (Qingdao, China), respectively. Tryptic Soy Broth (TSB) and Tryptic Soy Agar (TSA) for bacteria culturing were provided by Guangdong Huankai Microbial Sci. & Tech. Co., Ltd. (Guangdong, China). *A. hydrophila* used in this study was isolated and stored in our lab.

### 4.2. Preparation of Nanoemulsion

MSP and deionized water were prepared into aqueous phase (5% w:w) by stirring 3 h with the magnetic stirrer, in order to ensure MSP mixing. Then Linalool and MCT were prepared into oil phase (MCT: Linalool, 4:1, 3:2, 2:3, 1:4, and 0:5 v:v) by stirring 10 min with the magnetic stirrer. The following experimental steps were performed in an ice bath. To obtain Linalool colostrum, the aqueous phase was subsequently added to the oil phase 1:1 at 15,000 rpm in a high-speed homogenizer (F6/10, Shanghai Jingxin Industrial Development Co., Ltd., Shanghai, China). After homogenization for 5 min, the LN was prepared by ultrasonication (JY92-11D, Shanghai Jingxin Industrial Development Co., Ltd., Shanghai, China) at 700 w for 30 min [38].

#### 4.2.1. The Measurement of Droplet Size

The sample was diluted to a translucent and transparent concentration. Then the dynamic light scattering (DLS) test was used to measure the diameter of the droplet at 28 °C by Zetasizer Nano ZS90 (Malvern Instruments Co., Ltd., Malvern, UK).

#### 4.2.2. The Measurement of the Stability of Nanoemulsion and Colostrum

Nano-emulsion was distinguished from colostrum by a long-term thermodynamic stability test [23]. According to this method, nano-emulsion was transferred to a sterile brown glass vial for storage at 28 °C. The emulsion droplet size diameter of the samples was measured after 0, 4, and 10 d of storage, respectively.

#### 4.2.3. Fourier Transform Infrared (FTIR) Assay

To evaluate whether the chemical structure of Linalool was maintained during the preparation of LN, Linalool, the aqueous phase, and the nano-emulsion were chemically characterized by pressed-disk technique in an infrared spectrometer (Nicolet iS5, Thermo Fisher Scientific, Waltham, MA, USA).

#### 4.2.4. Observation of TEM

Firstly, the sample was diluted to an observable concentration. Then, an appropriate amount of diluent was dropped on the copper grid and phosphotungstic acid was added for negative stain. Finally, the morphology and size of LN were observed by a transmission electron microscope (HT-7700, Hitachi, Tokyo, Japan).

### 4.3. Antibacterial Activity Assay

#### 4.3.1. Determination of MIC and MBC

*A. hydrophila* was incubated overnight to logarithmic period, and sterile TSB was added to dilute the bacterial concentration to 1 × 10^6^ cfu/mL. LN were serially diluted by two-fold dilution in the concentration range of 10–0.30625%. Then, 100 μL of bacterial suspension and an equal amount of LN dilution were added to 96 well plate. Meanwhile, sterile TSB and bacterial suspension only were used as negative and positive controls, respectively. Finally, the samples were incubated at 28 °C with shaking (120 rpm). After 24 h incubation, the minimum concentration at which no visible growth of bacteria observed was MIC. For further determination of MBC, inoculating 10 μL of bacterial solution into sterile TSA, the minimum concentration at which no visible growth of bacteria colonies was determined as MBC.

#### 4.3.2. Fuorescence Microscope Assay

3 μL of SYTO^®^9 (Thermo Fisher Scientific, USA) were added to 1 mL of filtered sterile water to prepare a fluorescent staining dilution. Meanwhile, *A. hydrophila* was cultured in TSB until logarithmic phase, and then the supernatant was removed by centrifugation at 4000 rpm. Next, *A. hydrophila* were treated with 0 × MIC, 0.25 × MIC, 0.5 × MIC, and 1 × MIC of LN for 1 h. After washing two times with sterile PBS, 100 μL of fluorescent staining dilution was added to each sample, incubated for 30 min under light-proof condition and washed two times with sterile PBS. At last, 5 μL of the resuspension were used to make slides and observed under an inverted fluorescent microscope (OLYMPUS IX53, Olympus, Tokyo, Japan).

#### 4.3.3. Cell Activity Assay

1 × 10^8^ cfu/mL of overnight cultured *A. hydrophila* were collected by centrifugation at 4 °C for 10 min at 4000 rpm. Then the bacteria were re-suspended to 1 mL in sterile PBS, 0.5 × MIC, and 1 × MIC of LN, respectively. After that, the experimental groups were treated for 0.25 h, 0.5 h, 1 h, 1.5 h, and 2 h. Next, the bacterial solution was washed three times with sterile PBS and re-suspended to 1 mL of PBS. XTT labeling reagent and electron coupling reagent were mixed to prepare the stain, and then 100 μL of resuspension solution and 50 μL of XTT labeling mixture were added into a 96 well plate. After 4 h incubation at 28 °C, the absorbance of A_450_ values were measured by a microplate reader (Multiskan GO, Thermo Fisher Scientific, USA). The survival rate was calculated as below:

$$\mathrm{Survival}\;\mathrm{rate}\;(\%)=\frac{A_{450}\mathrm{Sample}}{A_{450}\mathrm{Control}}\times 100\%$$

#### 4.3.4. SEM Assay

*A. hydrophila* was grown in TSB until logarithmic phase. Then, the bacteria suspension was centrifuged at 4000 rpm, collected, and treated with 2×MIC of LN for 15 min, 30 min, and 60 min, respectively. The samples were washed twice with sterile PBS and fixed using 2.5% glutaraldehyde at 4 °C. After 4 h of fixation, the samples were centrifuged to remove glutaraldehyde and washed trice with sterile PBS. After that, the samples were dehydrated with 10%, 30%, 50%, 70%, and 90% ethanol for 15 min, followed by two dehydrations with 100% ethanol for 20 min each. Finally, the samples were replaced with tertiary butanol for 20 min and re-suspended with tertiary butanol. After freeze-drying and gold spraying, the samples were observed and photographed by a scanning electron microscope (Hitachi SU8010, Hitachi, Japan).

#### 4.3.5. TEM Assay

For TEM, the pre-treatment process was the same as mentioned in Section 4.3.4. The samples were washed with acetone, embedded in epoxy resin, ultra-thin sections, and stained with 1% phosphotungstic acid. Finally, the samples were observed by a transmission electron microscope (HT-7700, Hitachi, Japan).

### 4.4. Statistical Analysis

All experiments were carried out in triplicates. Statistical analysis was analyzed by using the Student’s *t*-test by GraphPad Prism 7, and *p* < 0.05 was considered to be significant and significant changes are indicated by asterisks in all figures.

## 5. Conclusions

In this study, nano-emulsion of Linalool was prepared to solve its low solubility and volatility, which is the obstacle for its application as aquatic drugs. The antibacterial activity and mode of action of LN against *A. hydrophila* were evaluated. Our results showed that LN were nanoscale spherical droplets and exhibited good long-term thermal stability and antimicrobial activity. The observation of SEM and TEM demonstrated that LN were able to destabilize the bacterial cell membrane, which indicated that the cell membrane may be a vital drug target of LN against *A. hydrophila*. In the future, multi-omics, such as transcriptomic, proteomics, and metabolomics may be performed to unravel the mode of action LN at gene and protein level.

## Data Availability

Not applicable.
